# Leveraging synthetic genetic array screening to identify therapeutic targets and inhibitors for combatting azole resistance in *Candida glabrata*

**DOI:** 10.1128/spectrum.02522-24

**Published:** 2025-08-11

**Authors:** Catrin C. Williams, Jane Usher

**Affiliations:** 1 Medical Research Council Centre for Medical Mycology, University of Exeter 3286, Exeter, United Kingdom; Universidade de Sao Paulo, Ribeirao Preto, Sao Paulo, Brazil

**Keywords:** *Candida glabrata*, antifungal resistance, genetic interactions, azole, SGA

## Abstract

Fungal infections affect over 6.55 million individuals annually, with *Candida glabrata* (recently reclassified as *Nakaseomyces glabratus*) being the second most common cause of invasive candidiasis and candidemia, conditions that carry a high mortality rate of approximately 63.6%. *C. glabrata* exhibits significant genomic plasticity, enabling rapid adaptation to antifungal treatments. This ability is largely driven by gain-of-function (GOF) mutations in the *CgPDR1* gene, which enhance the activity of efflux pumps and confer azole resistance. Over 80 unique *CgPDR1* mutations have been identified, leading to diverse resistance profiles, from single-azole resistance to pan-azole resistance. These mutations not only increase resistance but also enhance the virulence of *C. glabrata*. To combat multi-drug-resistant (MDR) strains, which show resistance to azoles, echinocandins, and polyenes, novel multi-target therapeutic approaches are essential. This study employs synthetic genetic array (SGA) screening to explore the genetic interactions underlying *CgPDR1*^+^-mediated azole resistance, using the clinical allele R592S. By generating genome-wide double mutant strains, we identified gene deletions that cause synthetic sick (SS) and synthetic lethal (SL) interactions, providing potential therapeutic targets. *In silico* screening identified inhibitors of these targets, and one was validated *in vitro*. Our findings support the potential of SGA screening to identify antifungal adjuvants that can slow the emergence of azole resistance in *C. glabrata*.

**IMPORTANCE:**

*Candida glabrata* is a significant cause of life-threatening fungal infections, especially concerning due to its rapid development of resistance to commonly used antifungal drugs like azoles. The growing prevalence of multi-drug-resistant *C. glabrata* strains, coupled with their high mortality rate, underscores the urgency of identifying new treatment strategies. This study investigates the genetic mechanisms underlying drug resistance, specifically focusing on mutations in the *CgPDR1* gene, which increase azole resistance. By leveraging a novel genetic screening method, this work identifies genes that interact with drug-resistant mutations, offering potential new therapeutic targets. Additionally, an inhibitor identified through *in silico* screening shows promise in delaying azole resistance when tested *in vitro*. The findings highlight the potential of multi-target therapies to combat drug resistance, paving the way for more effective treatment options for *C. glabrata* infections and addressing an urgent clinical need.

## INTRODUCTION

More than 6.55 million individuals are estimated to experience life-threatening fungal infections yearly (1). Of these, ~1.565 million individuals are thought to suffer invasive candidiasis or candidemia, which has an associated mortality rate of ~63.6% (2). *Candida glabrata (Nakaseomyces glabratus*) is the second most common causal agent of invasive candidiasis and candidemia (3, 4). *C. glabrata* belongs to the *Nakaseomyces* genus, possessing a haploid genome and displaying high genomic plasticity (5–11). The ability of *C. glabrata* to adapt and evolve in response to selective pressures, such as exposure to antifungal agents, underscores the importance of understanding its genetic makeup and the mechanisms underlying drug resistance. This genetic plasticity not only enables the emergence of resistance but also contributes to the persistence and spread of resistant strains within clinical settings (12).

Three main antifungal classes are used clinically: azoles, echinocandins, and polyenes (13, 14). Azoles, often the first line of treatment for *Candida* infections, are favored for their efficacy and relatively low toxicity (15). These drugs function by inhibiting lanosterol 14α-demethylase, an enzyme essential for the biosynthesis of ergosterol, a critical component of the fungal cell membrane (13, 16). Disruption of ergosterol synthesis compromises the integrity and function of the fungal membrane (13, 16). However, *C. glabrata* exhibits an intrinsic low-level tolerance to fluconazole (FLZ) and rapidly acquires high-level resistance to azoles (14, 17–22). This increasing resistance has led clinical guidelines, such as those from the IDSA, to recommend echinocandins as first-line therapies for treating invasive candidiasis (18, 23). Unfortunately, this shift has coincided with a rise in echinocandin-resistant *C. glabrata* infections, with certain regions reporting that up to 5% of *C. glabrata* isolates possess acquired resistance to echinocandins (14, 24–26). Moreover, multi-drug-resistant (MDR) *C. glabrata* strains have been documented, which are resistant to azoles, echinocandins, and polyenes (14). The rapid evolution of antifungal resistance in *C. glabrata* is a significant factor contributing to its high mortality rate (27). As conventional treatments become less effective, managing infections caused by this pathogen becomes increasingly challenging.

**Fig 1:**
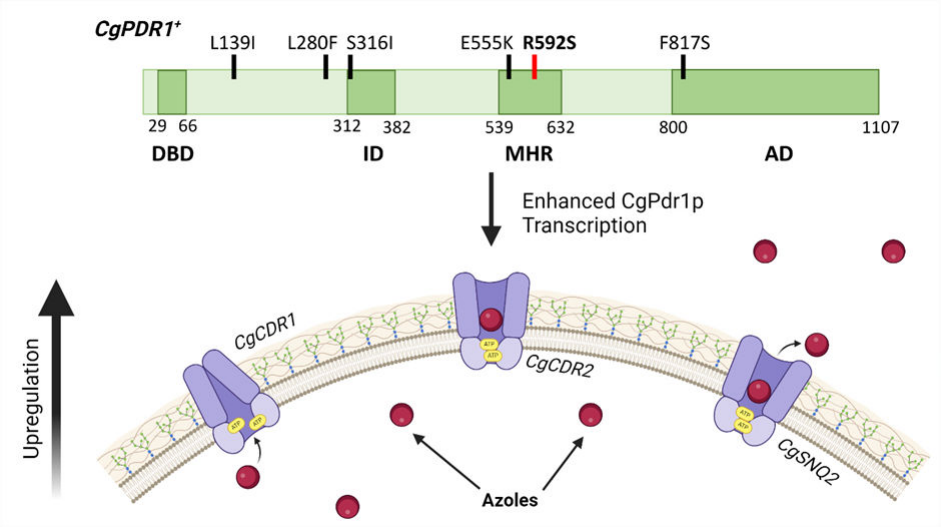
*CgPDR1^+^*-mediated azole resistance mechanism. Gain-of-function mutations within *CgPDR1* increase the expression of its encoded transcription factor, CgPdr1p. Increased CgPdr1p expression results in enhanced transcription of downstream genes such as *CgCDR1, CgCDR2,* and *CgSNQ2,* which encode efflux pumps. Dark green regions in the *CgPDR1* diagram represent coding regions: DNA-binding domain (+), inhibitory domain (ID), middle homology region (MHR), and transcriptional activation domain (AD). Light green regions in the diagram represent non-coding regions of the *CgPDR1* gene. The red band represents the *CgPDR1^R592S^* allele, which is assayed in this paper, whereas black bands represent *CgPDR1*^+^ alleles previously characterized. (Created with biorender.com).

High levels of azole resistance within *C. glabrata* are predominantly attributed to gain-of-function (GOF) mutations (*CgPDR1^+^*) within the *CgPDR1* (CAGL0A00451g) gene (22, 28–33). *CgPDR1^+^* leads to hyperactivity of the transcription factor CgPdr1p, increasing transcription of genes that encode drug efflux pumps such as *CgCDR1, CgCDR2,* and *CgSNQ2* (Fig. 1)*,* which belong to the ATP-binding cassette transporter family (3, 33–41). By increasing the expression of these efflux pump genes, *CgPDR1^+^* enhances the efflux of antifungal agents from the fungal cell, thereby reducing intracellular drug concentrations and rendering the fungus less susceptible to the effects of the drug (36, 40, 42, 43). This efflux-mediated resistance mechanism is a major contributor to the development of azole resistance in *C. glabrata* infections (36, 40, 42, 43). To date, more than 80 unique *CgPDR1^+^* have been described, which confer azole resistance (3, 29, 31, 32, 35, 37, 39, 40, 44–51). These GOF mutations involve amino acid substitutions in all domains of the *CgPDR1* gene: the transcriptional activation domain, the regulatory domain, the middle homology region (MHR), and non-domain regions (3, 29, 31, 32, 35, 37, 39, 40, 44–51) (Fig. 1). Unique *CgPDR1^+^* causes alternative expression profiles of efflux pumps, where one or more pumps can be upregulated (28, 32, 39, 40). Consistently, different mutations result in differing resistance profiles with some mutations conferring single azole resistance (e.g., FLZ only), whereas others allow pan-azole resistance (32, 36, 37, 39, 44–47, 50). Additionally, *C. glabrata* strains possessing *CgPDR1^+^* exhibit enhanced virulence, implying that evolution of drug resistance may increase the ability of *C. glabrata* to cause disease (39, 51).

The use of multi-target therapies is a well-established strategy to target drug resistance in numerous diseases ranging from cancer to malaria (3, 52–55). Targeting numerous components of the disease in combination is thought to slow the development of resistance, reducing toxicity and potential side effects (3, 52–55). Therefore, identification of novel multi-target therapies that slow the emergence of azole resistance is an attractive option for the treatment of the MDR pathogen *C. glabrata* (14). However, a major constraint to the identification of novel therapeutic targets is the need for previous characterization of gene function and how genes interact with a particular resistance mechanism (3). Previous studies have demonstrated that elucidation of the genetic interactions underlying *CgPDR1^+^*-mediated azole resistance, via synthetic genetic array (+) screening, allows for the identification of novel therapeutic targets in an unbiased and high-throughput manner (3, 56–58). In these screens, a *CgPDR1^+^* GOF allele is transformed and expressed in a *S. cerevisiae* strain, which is mated to a *S. cerevisiae* gene-deletion library, generating genome-wide double mutant strains (56, 59). This allows for the identification of gene deletions which cause synthetic sick (+) and synthetic lethal (SL) interactions in combination with a *CgPDR1^+^* allele (3). Therefore, elucidating genes required for the growth or survival of a *CgPDR1^+^*-expressing strain, respectively. A proof-of-concept study has previously shown that chemical inhibition of genes identified as causing a SL/SS interaction with *CgPDR1^+^* alleles via SGA slows the emergence of azole resistance within *C. glabrata* strains (3). However, this study utilized an inhibitor that is toxic to mammalian cells (3, 60).

In this study, we performed an SGA screen of a clinical CgPDR1 + allele R592S from a clinical isolate with an MIC50 of 32 µg/mL to Fluconazole. This approach allowed us to construct a more comprehensive genetic interaction map for multiple GOF alleles of varying clinical severity. Additionally, we identified potential common therapeutic targets, screened for inhibitors of target genes via *in silico* analysis, and validated a promising inhibitor *in vitro*. Ultimately, this study aims to demonstrate the utility of SGA in identifying medically relevant antifungal adjuvants, which can be used alongside existing treatments to slow the emergence of azole resistance in C. glabrata.

## MATERIALS AND METHODS

### Strains used in this study

Table 1 lists the strain used in this work.

**TABLE 1:** Strains used in this work

Strain name	GOF mutation	*PDR1* domain	FLZ MIC (µg/mL)	Origin
BG2 (*C. glabrata*)	–^*b*^	–	^*a*^	This study
CBS138 (*C. glabrata*)	–	–	^*a*^	This study
B8441 (*C. auris*)	–	–	^*a*^	Gift from Dr. Duncan Wilson
SC5314 (*C. albicans*)	–	–	2	This study
CD34 (*C. dubliniensis*)	–	–	^*a*^	Sullivan 1997
CDC317 (*C. parapsilosis*)	–	–	2	(61)
MYA-3404 (*C. tropicalis*)	–	–	2	Gift from Dr. Duncan Wilson
DSY2277	R592S	MHR	32	(39)
DSY2268	S3161I	INHIBITORY	32	(39)
DSY3638	E555K	MHR	128	(39)
DSY3675	F817S	ACTIVATION	128	(39)
DSY565	L280F	–	128	(35)
DSY2258	L139I	–	128	(37)

^*a*^ no MIC has been determined using guidelines as determined by EUCAST.

^*b*^ "–” indicates not applicable.

### Synthetic genetic array (SGA) screening

Wild-type *CgPDR1* and gain-of-function *CgPDR1^R592S^* alleles were amplified via PCR and sequence verified. Subsequently, Gateway cloning was used for plasmid generation (56, 62). Standard LiAc transformation was performed to transform the pDEST plasmids (containing a *URA3* selection marker) into the *S. cerevisiae* strain Y7092 (3, 63, 64).

To perform the SGA screen, the Singer RoTor HAD (Singer Instruments) was used to manipulate the deletion mutant array (DMA), as described by Usher and Haynes (2019) (3). During SGA screening, specific agar is used for each step of the screen (Table S1). Haploid *S. cerevisiae* MATα strains expressed either pDEST426-ccdB-GPD-CgPDR1 or pDEST426-ccdB-GPD- CgPDR1^R592S^. Haploid MATα strains were then mated to a haploid MATa DMA (Invitrogen Yeast Deletion MAT, Complete Set, 10175114), and diploid strains that expressed a *CgPDR1* allele and a single gene deletion were selected for, as detailed by Usher et al*.* (58). Robot plates were imaged using PhenoBooth+ (Singer Instruments). The SGA screen was analyzed to identify SL (synthetic lethal), SS (synthetic sick), or suppression (SUP) phenotypes. SUP phenotypes refer to increased growth in strains that express both a *CgPDR1* allele and a single gene deletion. The entire screen was performed in triplicate; phenotypes were only recorded as a genetic interaction if they were shared by 2–3 replicates.

### Data visualization and statistical analysis

To analyze genetic interaction data sets, the FunSpec (http://funspec.med.utoronto.ca/) database was utilized. FunSpec performs a hypergeometric distribution test to provide *P*-values utilizing a Bonferroni correction to determine which genes are enriched within the data set (where *P* < 0.01) and provides information about the function and localization of genes. The data visualization software, Osprey (v.1.2.0), was used to render genetic interaction maps of genes that were enriched within the data set.

### Inhibitor identification

#### *In silico* inhibitor identification

To identify the potential inhibitors of genes of interest *in silico,* the inhibitor database HIP HOP was utilized (https://hiphop.fmi.ch/). The Z-score produced by HIP HOP for each compound was used as an indicator of how well the compound would theoretically inhibit the gene. HIP HOP calculates inhibitor Z-scores by dividing the sensitivity score by the standard deviation, thus providing a measurement of the distance between the sensitivity score of an inhibitor of interest and the mean sensitivity score (65).

### *In vitro* inhibitor validation

#### Spot assays

For all antifungal and inhibitor screens, WT strains (BG2 and CBS138) and clinical isolates (Table 1) were assayed. Strains were grown overnight in CSM broth at 30°C and 200 rpm. Serial dilutions of cultures were prepared, with the final inoculum concentrations ranging from 10^6^ to 10^2^ cells/mL; 5 µL of inoculum was spotted onto CSM agar plates, and all agar conditions are detailed in Table 2. Plates were incubated at 30°C and imaged at 24 h and 48 h using the PhenoBooth+. CSM only and CSM agar containing DMSO were used as growth controls. For CSM plates containing FLZ, the MIC_50_ of WT strains and clinical isolates was assayed. The MTX concentrations used were the lowest concentrations reported by Yang et al*.* (66). The combinatorial therapies utilized were guided by findings from previous Fluconazole-only (FLZ) and Methotrexate-only (MTX) screens.

**TABLE 2:** Agar conditions used in spot assays

Controls	FLZ only	MTX only	FLZ & MTX combi
CSM only	8 µg/mL	0.04 mg/mL	FLZ (8 µg/mL) & MTX (0.04 mg/mL)
CSM + DMSO	32 µg/mL	0.08 mg/mL	FLZ (8 µg/mL) & MTX (0.08 mg/mL)
	128 µg/mL	0.16 mg/mL	FLZ (8 µg/mL) & MTX (0.16 mg/mL)
		0.32 mg/mL	FLZ (8 µg/mL) & MTX (0.32 mg/mL)

#### MTX and FLZ checkerboard screening

Potential synergy of MTX and FLZ was investigated using checkerboard screening. Strains were grown in CSM broth overnight at 30°C and 200 rpm. For all checkerboard screens, a final concentration of 1.75 × 10^5^ cells/mL was used in keeping with recommended EUCAST antifungal susceptibility testing guidelines (67). Checkerboard screens also had inbuilt sterility controls (media and ddH_2_O) and growth controls (media and two times inoculum). Isolates were exposed to a single stress of both drugs independently at a range of concentrations and in all possible combinations to assess the potential synergy of the drugs. For high-dose screens, efficacy of FLZ at 4 µg/mL—256 µg/mL and MTX at 0.04 mg/mL—10.24 mg/mL against isolates was assessed as a single stress and as combinatorial stresses (Fig. S1). Low-dose screens assayed FLZ at 4 –256 µg/mL and MTX at 0.0025 –1.28 mg/mL as single and combinatorial stresses. Plates were incubated at 37°C for 48 h, with OD_530_ measurements taken using a Tecan Spark at 0 h and 24 h. Heatmaps displaying relative growth of isolates were generated using RStudio, where isolate growth is scored from zero in black, to one in bright green (code provided in Supplemental Methods 1). To assess synergy, fractional inhibitory concentration index (FICI) values were calculated (Equation 1). In concordance with John et al*.*, FICI values ≤ 0.5 were classed as synergistic, >0.5 and <1.0 were partially synergistic, 1.0 was additive, and >1 and <4 were indifferent, and values > 4 were considered antagonistic (68). FICI values were then overlaid onto heatmaps, as shown in Liston et al*.* (69).


$$FICI=\frac{MIC50drugAcombination}{MIC50drugAalone}+\frac{MIC50drugBcombination}{MIC50drugBalone}$$

### *Galleria mellonella* toxicity screening

To determine if there was any associated toxicity to MTX, screening using *Galleria mellonella* (UK waxworms, Sheffield) was performed. Larvae were injected with either 5 µL of DMSO (control group) or MTX. For each condition, 10 larvae were injected per replicate, with a total of three replicates performed. Several concentrations of MTX were assayed: 0.04 mg/mL, 0.08 mg/mL, 0.32 mg/mL, 2.56 mg/mL, 5.12 mg/mL, and 10.24 mg/mL. Larvae were incubated at 37°C for 6 days, with survival recorded daily. A log-rank (Mantel-Cox) test was performed to assess if there was a significant difference in the survival of larvae, where *P* < 0.05. Analysis and visualization of Kaplan-Meier survival curves was performed using PRISM (v.10.2.1).

### Quantitative real-time reverse-transcription-PCR

RNA was prepared from cells following either no drug exposure, MTX exposure only, FLZ exposure only, or MTX and FLZ (combination) exposure, using the hot-phenol-chloroform method. cDNA was synthesized from 2 µg RNA using the ImProm-II reverse transcriptase kit (Promega UK) per the manufacturer’s instructions; 1 µL cDNA was used for each SYBR gene reaction (SYBR green PCR master mix: Qiagen, UK). Transcript levels were normalized using Quant Studio Design and Analysis Software (Applied Biosystems, version 2.8.0) against the internal *PDA1* mRNA controls.

## RESULTS

### Identification of the genetic interaction networks, which underlie azole resistance within *C. glabrata*

The most common mechanism of azole resistance within *C. glabrata* is via GOF mutations within the *CgPDR1* gene (*CgPDR1^+^*), which causes increased expression of CgPdr1p and upregulation of downstream genes such as *CgCDR1, CgCDR2,* and *CgSNQ2,* which encode efflux pumps (Fig. 1) (3, 33–41).

**Fig 2:**
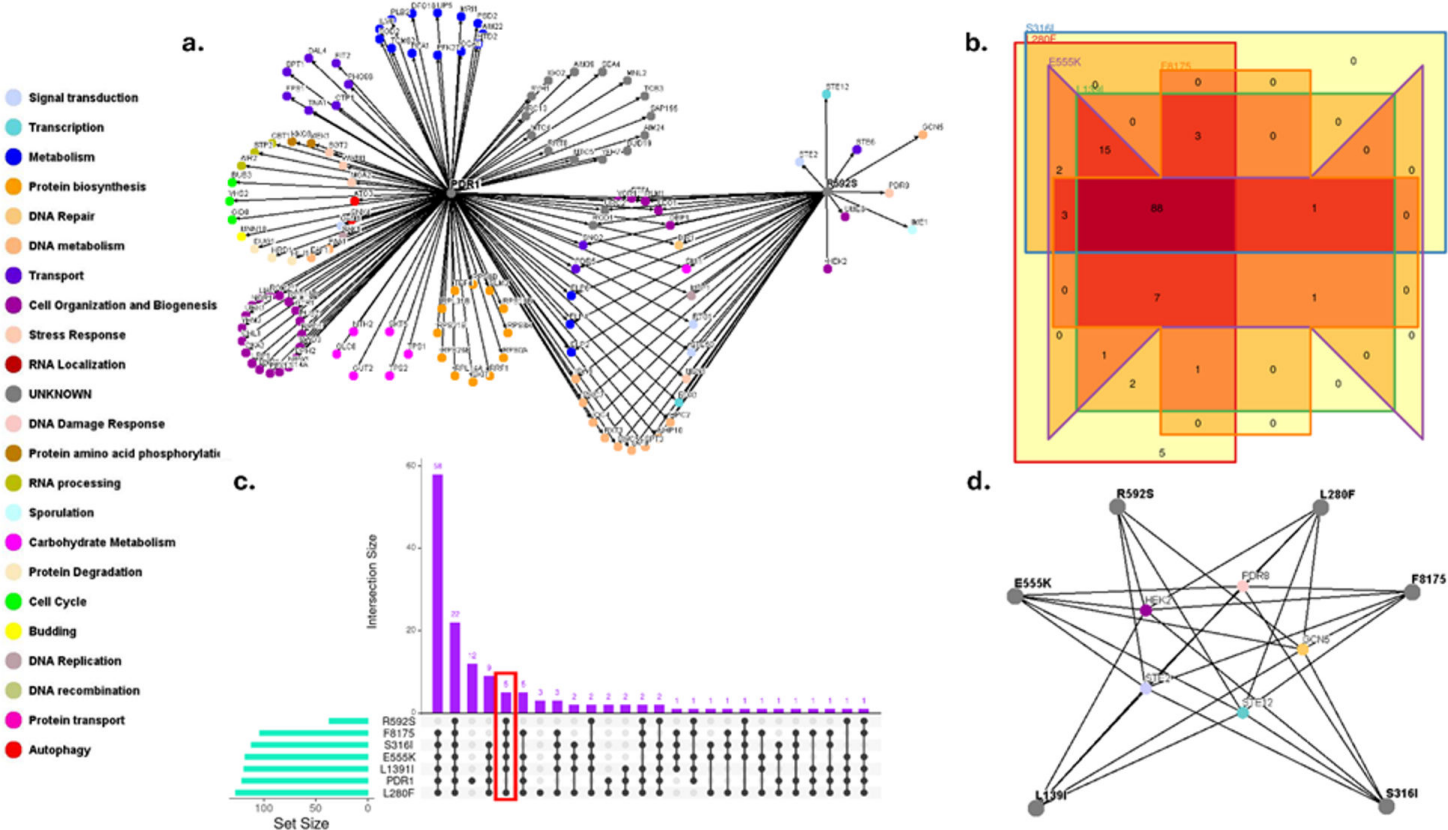
Identifying genetic interaction networks underlying *C. glabrata* azole resistance. (**a**) A genetic interaction map of genes, which, when deleted, caused a SL or SS phenotype in combination with a WT *CgPDR1* allele and/or *CgPDR1^R592S^* allele. *CgPDR1* and *CgPDR1^R592S^* alleles are represented by the gray hubs; each node represents a single gene deletion, which is necessary for the survival or growth of *S. cerevisiae* strains expressing a *CgPDR1* or *CgPDR1^R592S^* allele. Nodes are colored by gene function; their GO terms are listed on the left-hand side. (**b**) A battle Venn diagram of the five SGA screens of different *CgPDR1* GOF alleles, which have been previously performed. These screens include *CgPDR1^S316I^* (blue), *CgPDR1^F8175^* (red), *CgPDR1^E555K^* (purple), *CgPDR1^L280F^* (orange), and *CgPDR1^L139I^* (green). These GOF mutations occur in the inhibitory domain, activation domain, middle-homology region, and non-coding regions (*CgPDR1^L280F^* & *CgPDR1^L139I^*), respectively. This battle Venn diagram shows that unique genetic interaction profiles are possessed by distinct *CgPDR1^+^* +. (**c**) An upset plot which compares the *CgPDR1* WT and *CgPDR1^R592S^* allele with all other SGAs performed. Notably, there are five interactions that are shared by all *CgPDR1*^+^ alleles, as highlighted in red. These five interactions are unique to *CgPDR1*^+^ alleles and are not shared by WT *CgPDR1*. (**d**) A genetic interaction map displaying the function of the five genes, which, when deleted, cause a SL and SS interaction with all *CgPDR1*^+^ alleles. Each *CgPDR1^+^* + is displayed in gray hubs, and the GO terms of these genes are displayed on the left-hand side.

An SGA screen was performed with a *S. cerevisiae* strain expressing a WT *CgPDR1* allele and a strain expressing a *CgPDR1^R592S^* allele, which was isolated from a FLZ-resistant clinical isolate (Table 1). The screen identified genes that resulted in a SL or SS interaction in combination with a *CgPDR1* and/or a *CgPDR1^R592S^* allele. Gene enrichment analysis of SL and SS interactions was determined by a hypergeometric distribution test performed via the FunSpec database (*P* < 0.01). This analysis provided GO annotations for genes that were enriched within the data set. A genetic interaction map of these SS and SL interactions was plotted (Fig. 2a). There were 93 unique SL and SS interactions to the *CgPDR1* allele, 29 interactions were common to both alleles, and eight interactions were unique to the *CgPDR1^R592S^* allele (Table S2; Fig. S1). There was an enrichment of genes involved in transcription, chromatin remodeling, and ATPase activity.

Different *CgPDR1^+^* results in unique azole susceptibility profiles with certain GOF mutations conferring resistance to a single azole, whereas others confer triazole resistance (32, 36, 37, 39, 44–47, 50). Unique *CgPDR1^+^* can also result in differential upregulation of single or multiple efflux pumps (28, 32, 39, 40). Consistently, previous SGA screens showed that different *CgPDR1^+^* possessed unique genetic interaction profiles (Fig. 2b). These screens included the alleles *CgPDR1^S316I^, CgPDR1^F8175^, CgPDR1^E555K^, CgPDR1^L280F^*, and *CgPDR1^L139I^*, which possessed mutations in the inhibitor domain, activation domain, middle-homology region, and non-domain regions (*CgPDR1^L280F^* and *CgPDR1^L139I^*) of *CgPDR1,* respectively (Fig. 1). These previous SGA screens were then compared with the *CgPDR1* and *CgPDR1^R592S^* alleles (Fig. 2c). FunSpec analysis showed significant enrichment of genes involved in transcription (*UME6*), chromatin remodeling (*GCN5*), and ATPase activity (*SNQ2* and *YOR1,* which encode efflux pumps). There were 22 interactions, which were shared by *CgPDR1* and all *CgPDR1^+^* (Table S2).

There were five genes that were common to all *CgPDR1^+^* (Fig. 2c and d). These five genes, *HEK2, PDR8, GCN5, STE2,* and *STE12,* therefore represent promising potential therapeutic targets as they exclusively interact with *CgPDR1^+^*, which confers azole resistance (Fig. 2d). *HEK2* is involved in mRNA localization, mRNA stabilization, and telomere maintenance (70–75). *PDR8* encodes the transcription factor Pdr8p, which, similar to Pdr1p, targets the ABC and MFS transporters (76–79). However, there is no *PDR8* ortholog in *C. glabrata* and thus does not represent a good potential therapeutic target. *GCN5* is involved in chromatin remodeling; it is a catalytic subunit of the ADA and SAGA histone acetyltransferase complexes (80–87). The α-factor pheromone receptor, *STE2,* triggers the signaling cascade, which allows for mating between haploid mat-a and mat-α cells (88–90). Finally, *STE12* encodes a transcription factor, which is involved in mating alongside pseudohyphal and hyphal growth (91–93). *GCN5* was selected as the gene target of interest as its ortholog, *CgGCN5,* has previously been implicated in azole resistance within *C. glabrata* (94).

### There is potential to repurpose existing therapeutics as inhibitors of genes, which causes SL or SS interactions with *CgPDR1*^+^ mutations

**Fig 3:**
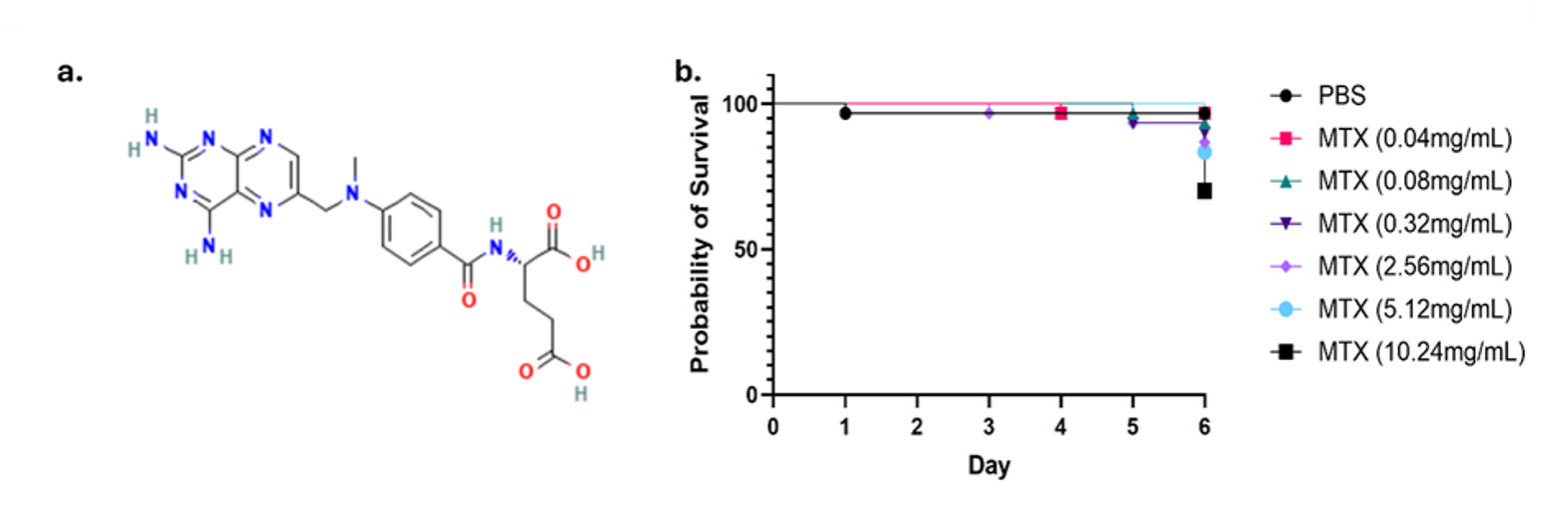
Identification of potential inhibitors of *GCN5.* (**a**) The chemical structure of Methotrexate (MTX) was identified via *in silico* analysis as a promising potential inhibitor of *GCN5*. (Image from PubChem.com). (**b**) Potential toxicity of MTX was investigated using the model organism, *Galleria mellonella*. Larvae were injected with 0.04 mg/mL, 0.08 mg/mL, 0.32 mg/mL, 2.56 mg/mL, 5.12 mg/mL, and 10.24 mg/mL of MTX alongside PBS as a control group. Survival of larvae over 6 days is displayed on the Kaplan-Meier survival curve, which shows that MTX displayed low toxicity in *G. mellonella*.

*In silico* identification of inhibitors was performed utilizing the HIP HOP database. Methotrexate (MTX) was identified as a potential inhibitor of *CgGCN5* (Fig. 3a). MTX is an FDA-approved immunosuppressant utilized to treat diseases such as rheumatoid arthritis (95). MTX is therefore expected to display low levels of toxicity. To confirm this, a toxicity screen was performed in the model organism *Galleria mellonella* (Fig. 3b). MTX demonstrated low levels of toxicity in this model at all doses tested, with most death occurring at day 6 in a dose-dependent manner. There was a significant difference, where *P* = 0.0343, between the survival of larvae injected with 10.24 mg/mL of MTX. However, all other doses of MTX did not significantly affect the survival of larvae compared to larvae injected with PBS (*P* > 0.05).

### MTX displayed potential synergy with FLZ against *C. glabrata*

**Fig 4:**
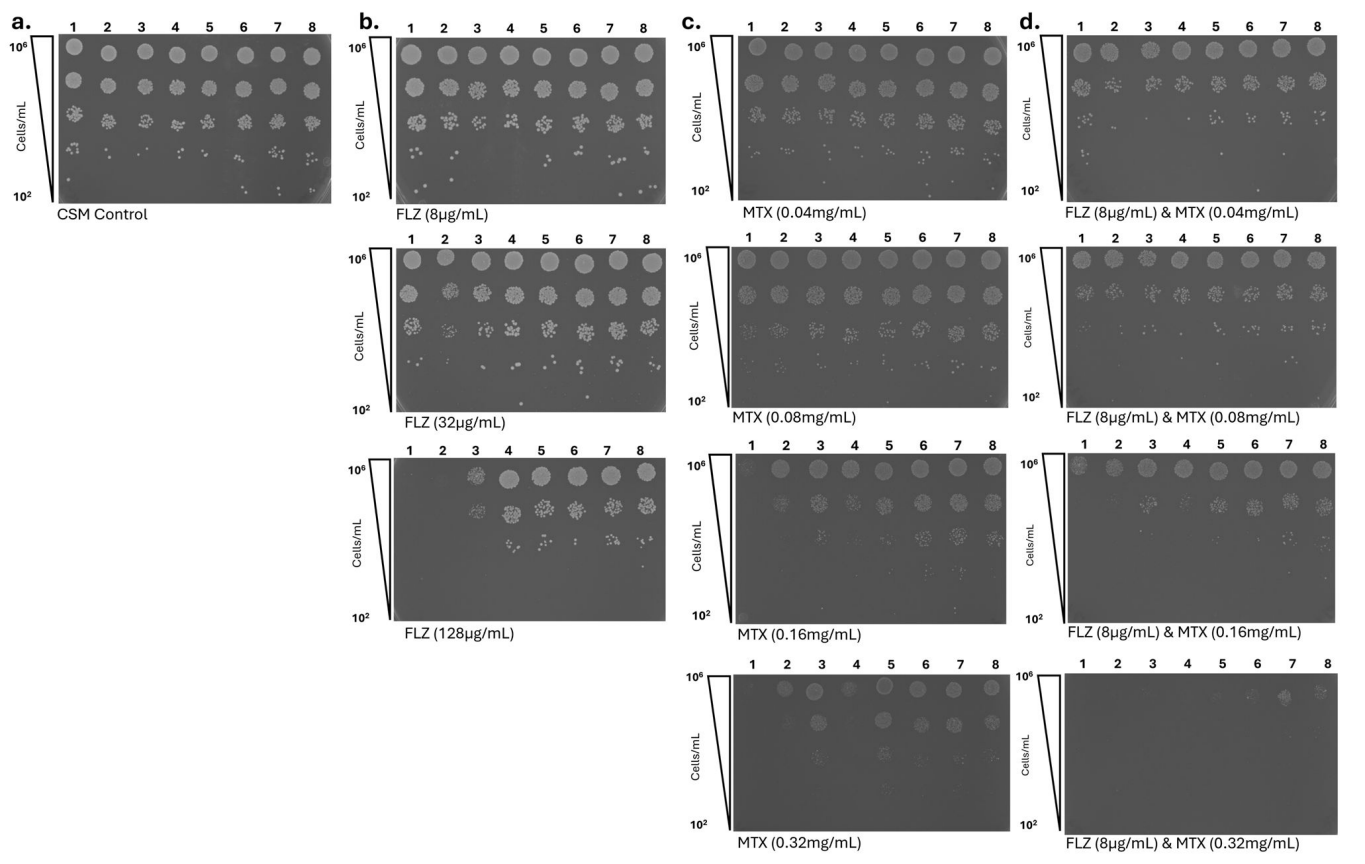
Preliminary validation of MTX *in vitro,* as a single and combinatorial therapy. Spot assays show the growth of eight *C. glabrata* strains; strains one and two are *C. glabrata* wild-type strains BG2 and CBS138, strains 3–8 are clinical isolates, which possess *CgPDR1^R592S^, CgPDR1^S316I^, CgPDR1^E555K^, CgPDR1^L280F^, CgPDR1^L139I^*, and *CgPDR1^F817S^* alleles, respectively. (**a**). Growth of strains after 24 h on CSM minimal media alone. (**b**) Strains were grown on 8 µg/mL, 32 µg/mL, and 128 µg/mL of FLZ for 24 h. Inhibition of growth of WT strains is seen at 32 µg/mL, whereas slight inhibition of growth of clinical isolates is seen at 128 µg/mL FLZ. (**c)** Growth of strains on CSM agar containing 0.04 mg/mL, 0.08 mg/mL, 0.16 mg/mL, and 0.32 mg/mL of MTX after 24 h. This shows 0.32 mg/mL of MTX inhibits growth of all strains. (**d**) To investigate the potential synergy of MTX with FLZ, strains were grown on 8 µg/mL of Fluconazole & 0.08 mg/mL, 0.16 mg/mL, and 0.32 mg/mL of MTX, respectively. Inhibition of growth was seen at 0.16 mg/mL and 0.32 mg/mL of MTX in combination with 8 µg/mL of FLZ.

To assess whether MTX could replicate the SL/SS interaction with *CgPDR1^+^*, preliminary *in vitro* validation of MTX was performed. *C. glabrata* WT strains, BG2 and CBS138, were tested alongside clinical isolates, which possess *CgPDR1^R592S^, CgPDR1^S316I^, CgPDR1^E555K^, CgPDR1^L280F^, CgPDR1^L139I^*, and *CgPDR1^F817S^* GOF mutations (Fig. 1 and 4). Spot assays on CSM agar showed normal growth of all strains (Fig. 4a). To determine azole susceptibility profiles of these strains, all strains were grown at 8 µg/mL, 32 µg/mL, and 128 µg/mL of FLZ (Fig. 4b). As expected, growth of the WT *C. glabrata* strains was inhibited at 8 µg/mL of FLZ, which is the MIC_50_ for *C. glabrata* isolates. Consistent with previously published MIC_50_ data for the clinical isolates assayed, isolates possessing *CgPDR1*^R592S^ and *CgPDR1^S316I^* alleles showed partial inhibition of growth at 32 µg/mL of FLZ. Although isolates possessing *CgPDR1^E555K^, CgPDR1^L280F^, CgPDR1^L139I^, *and *CgPDR1^F817S^* alleles only showed inhibition of growth at ≥128 µg/mL of FLZ. Growth on low concentrations of MTX was tested; 0.04 mg/mL, 0.08 mg/mL, 0.16 mg/mL, and 0.32 mg/mL (Fig. 4c). Initial inhibition of growth was observed at 0.04 mg/mL of MTX, whereas growth of all strains was severely inhibited at 0.32 mg/mL of MTX (Fig. 4c). To investigate whether there was potential synergy between MTX and FLZ, isolates were grown on agar containing 8 µg/mL of FLZ in combination with low concentrations of MTX (0.04 mg/mL, 0.08 mg/mL, 0.16 mg/mL, and 0.32 mg/mL) (Fig. 4d). Slight inhibition of growth was seen at FLZ 8 µg/mL in combination with MTX 0.04 mg/mL and MTX 0.08 mg/mL, whereas strong inhibition of growth was seen at FLZ 8 µg/mL and MTX at both 0.16 mg/mL and 0.32 mg/mL.

**Fig 5:**
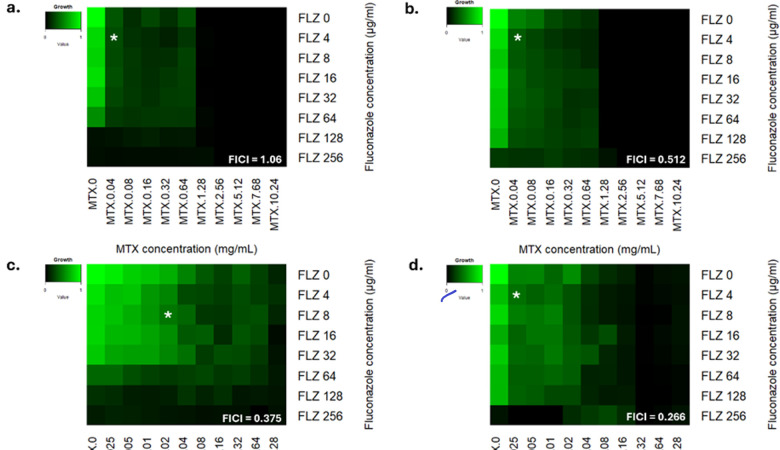
Assessing efficacy of combinatorial MTX & FLZ therapies. Heatmap displaying the relative growth of (**a**) *C. glabrata* WT strain BG2 and (**b**) a clinical isolate possessing the *CgPDR1*^R592S^ in various concentrations of Fluconazole, ranging from 8 to 256 µg/mL, and Methotrexate, ranging from 0.04 to 10.24 mg/mL. Lowest drug concentrations are at the top left of the heatmap, and the highest drug concentrations are at the bottom right of the heatmap. Growth of strains is scored from 0 (black) to 1 (green). Growth of both strains is completely inhibited at ≥1.28 mg/mL of MTX alone or in combination with any concentration of Fluconazole. The fractional inhibitory concentration index (FICI) value is indicated by an overlaid white asterisk, with the value indicated in the bottom right-hand side of heatmaps. Note, FICI values displayed are calculated for the MIC_50_ of drugs. Relative growth of *C. glabrata* isolates, (**c**) BG2, and (**d**) a clinical isolate possessing the *CgPDR1*^R592S^ allele, in a range of FLZ concentrations from 8 to 256 µg/mL and MTX concentrations from 0.0025 to 1.28 mg/mL. The FICI value is indicated by an overlaid white asterisk, with values printed in the bottom right-hand side of heatmaps. FICI values displayed are calculated for MIC_50_ of drugs. All checkerboards were performed in triplicate: heatmaps display the average relative growth of isolates, and FICI values were calculated for average growth. Checkerboard screening was repeated an additional two times, with similar results obtained.

To determine if there was synergy between MTX and FLZ, checkerboard assays were performed. Growth of BG2 (WT strain) and a clinical isolate possessing *CgPDR1^R592S^* allele was assayed in single concentrations of MTX (0–10.24mg/mL) and FLZ (0–256µg/mL). Growth of isolates was also assessed in combinations of each MTX and FLZ concentration (Fig. S1). A heatmap assessing relative growth of BG2 (Fig. 5a) and a clinical isolate possessing the *CgPDR1^R592S^* allele was produced (Fig. 5b). As expected, the isolate possessing the *CgPDR1^+^* was able to grow in higher concentrations of FLZ alone compared to BG2. This heatmap shows that MTX is effective at reducing the growth of *C. glabrata* at all concentrations tested. Notably, growth of both strains was completely inhibited at ≥1.28 mg/mL of MTX alone and in combination with any FLZ concentration. This assay showed that MTX and FLZ combinatorial therapy had a partially synergistic effect against the clinical isolate possessing a *CgPDR1^R592S^* allele, whereas combinatorial therapy had an indifferent effect against BG2 (Fig. 5a and b). However, the lowest concentration of MTX tested in combination with the lowest concentration of FLZ only allowed for ~30% growth of isolates. Therefore, a further checkerboard assay of lower concentrations of MTX was undertaken (Fig. 5c and d). Single concentrations of MTX (0 –1.28 mg/mL) and FLZ (0–256 µg/mL) and relevant combinations of both drugs were screened (Fig. 5c and d; Fig. S2). These checkerboards displayed a more discreet variation in the inhibition of isolate growth. Combinatorial MTX and FLZ therapy had a synergistic effect on both BG2 and a clinical isolate possessing the *CgPDR1^R592S^* allele, as indicated by FICI values < 0.5 (Fig. 5c and d). Similar to initial checkerboards (Fig. 5a and b), almost complete inhibition of growth of BG2 and an isolate harboring a *CgPDR1^R592S^* allele was observed at 1.28 mg/mL of MTX alone or in combination with any concentration of FLZ (Fig. 5c and d). BG2 was more sensitive to FLZ stress than the isolate possessing a *CgPDR1^R592S^* allele. However, BG2 was more tolerant of MTX stress (displaying significant growth in ≥0.64 mg/mL MTX) compared with the isolate, ranging from 8 to 256 µg/mL and Methotrexate ranging, which possessed a *CgPDR1^R592S^* allele (which only displayed significant growth in ≥0.16 mg/mL MTX). Overall, increasing concentrations of FLZ and MTX in combination more effectively inhibited the growth of *C. glabrata* (Fig. 5).

### Gene expression analysis

To evaluate gene expression levels, quantitative PCR (Q-PCR) was performed on both wild-type and DSY227 (*CgPDR1^R592S^*) cells under four distinct conditions: no treatment, methotrexate (MTX) alone, fluconazole (FLZ) alone, and a combination of MTX and FLZ (combi). Gene expression was quantified using the relative quantification (RQ) method, with untreated wild-type cells serving as the control. The target genes analyzed included *GCN5, STE12, STE2, HEK2*, and *PDR1*, whereas *PDA1* was used as the reference gene. The RQ values represent the fold change in gene expression relative to the control condition, calculated using the ΔΔCt method.

The results (Fig. S3) indicate that in wild-type cells, MTX treatment alone led to an upregulation of *GCN5* compared with the untreated control. In contrast, FLZ treatment resulted in a significant increase in *PDR1* expression, whereas the expression levels of other genes remained unchanged. Notably, when wild-type cells were exposed to the combination of MTX and FLZ, no significant changes were observed in the expression of any of the examined genes.

In DSY227 (*CgPDR1^R592S^*) cells, basal conditions (no drug exposure) were associated with elevated *PDR1* expression relative to wild-type cells. When treated with MTX alone, these mutant cells exhibited a significant upregulation of *GCN5* and *STE2*, accompanied by a decrease in *PDR1* expression. Similarly, FLZ exposure resulted in increased expression of *GCN5, STE2*, and *STE12*, whereas *PDR1* expression was downregulated. Under combinatorial treatment with MTX and FLZ, *GCN5* and *STE2* expressions remained elevated, whereas *PDR1* expression was consistently reduced relative to wild-type cells. These findings highlight distinct gene expression responses in wild-type and mutant *C. glabrata* strains, with the *CgPDR1R592S* mutation influencing transcriptional adaptations to antifungal and chemotherapeutic agents.

### Screening of other *Candida* species on FLZ and MTX

**Fig 6:**
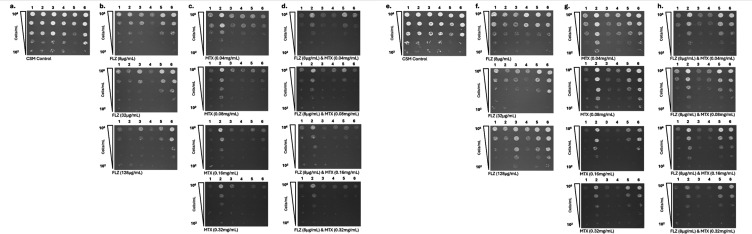
Spot assays to determine the efficacy of Fluconazole and Methotrexate on other *Candida* species. Spot assays were performed on a sub-collection of *Candida* species at both 30°C (**a-d**) and 37°C (**e-h**) in the presence of FLZ and MTX alone and in combination with FLZ (8 µg/mL) and increasing concentrations of MTX (range 0.04–0.32 mg/mL). The *Candida* species tested were 1: *C. glabrata* (BG2); 2: *C. auris* (type strain B8441); 3: *C. albicans* (5314); 4: *C. dubliniensis* (type strain CD36); 5: *C. tropicalis* (type strain MYA-3404), and 6: *C. parapsilosis* (type strain CDC317). Strains were grown on CSM (**a** & **e**) without the presence of an antifungal drug as a control. Panels (**b** and **f**) show increases in FLZ exposures ranging from 8 µg/mL to 128 µg/mL. Panels (**c and g**) show growth on increasing concentrations of MTX ranging from 0.04 mg/mL to 0.32 mg/mL. Panels (**d and h**) show growth on media containing a combination of FLZ at 8 µg/mL and increasing concentrations of MTX.

Finally, we wanted to determine if there was efficacy of MTX, singly and in combination with FLZ, against other *Candida* species. We performed a series of spot assays on increasing concentrations of FLZ, MTX, and FLZ and MTX on *C. albicans*, *C. auris*, *C. dubliniensis*, *C. tropicalis,* and *C. parapsilosis* at both 30°C and 37°C (Fig. 6a through h). As expected from MIC data, there was no observed inhibition of growth on the lower concentrations of FLZ at both temperatures; at 32 µg/mL, we observed inhibition of growth of *C. glabrata, C. albicans,* and *C. dubliniensis *(17, 61, 67, 96). It is not until the higher concentration of 128 µg/mL that we observed inhibition of all strains. On plates containing only increasing concentrations of MTX, we observed that all strains except for *C. albicans* showed inhibition of growth, until 0.32 µg/mL when *C. albicans* growth is inhibited (Fig. 6c). For the combination of FLZ and MTX, we observed inhibition of growth of all strains at 8 µg/mL FLZ and 0.16 mg/mL MTX at 30°C, whereas at 37°C, we observed inhibition at the higher MTX concentration.

## DISCUSSION

The increasing incidence of antifungal resistance among *C. glabrata* strains poses a significant challenge in clinical settings (97). This study aimed to identify novel therapeutic strategies to combat this issue by targeting genetic interactions involved in azole resistance, specifically those mediated by gain-of-function (GOF) mutations in the *CgPDR1* gene (*CgPDR1^+^*). The results from this study provide valuable insights into potential multi-target therapies that could be used to slow the emergence of azole resistance and enhance treatment efficacy.

### SGA screening

The results of this study highlight the complexity of azole resistance mechanisms in *C. glabrata*, particularly the role of gain-of-function mutations in the *CgPDR1* gene. These mutations lead to the overexpression of efflux pumps, including *CgCDR1*, *CgCDR2,* and *CgSNQ2*, which significantly contribute to azole resistance (3, 33–41). By performing an SGA screen, we have identified multiple genes that demonstrate synthetic lethal or synthetic sick phenotypes when combined with *CgPDR1* and *CgPDR1^R592S^* alleles. The genetic interaction map (Fig. 2a) revealed that 93 unique SL and SS interactions are specific to the *CgPDR1* allele, with 29 interactions shared by both alleles and eight unique to the *CgPDR1^R592S^* allele. There was an enrichment of genes involved in transcription, chromatin remodeling, and ATPase activity, which may provide novel therapeutic targets for overcoming azole resistance. Our findings corroborate previous research, which has shown that different *CgPDR1^+^* results in unique azole susceptibility profiles (32, 36, 37, 39, 44–47, 50). Some GOF mutations confer resistance to a specific azole, whereas others provide broader resistance to triazoles (32, 36, 37, 39, 44–47, 50). Additionally, different *CgPDR1^+^* lead to the differential upregulation of single or multiple efflux pumps, further complicating the resistance profile (28, 32, 39, 40). The uniqueness of the genetic interaction profiles of various *CgPDR1^+^*, including *CgPDR1^S316I^, CgPDR1^F817S^*, *CgPDR1^E555K^*, *CgPDR1^L280F^*, and *CgPDR1^L139I^*, as observed in our and previous SGA screens (Fig. 2b and c), suggests that targeted therapeutic strategies should consider the specific *CgPDR1^+^* mutation present.

Interestingly, five genes (*HEK2*, *PDR8*, *GCN5*, *STE2*, and *STE12*) were common to all *CgPDR1^+^*, suggesting that they are critical for the *CgPDR1*-mediated azole resistance mechanism. Of these, only *PDR8* does not possess an orthologous gene in *C. glabrata* (98). This limits the potential of targeting *PDR8* to slow the emergence of resistance in *C. glabrata*. In addition, *PDR8* encodes a transcription factor, Pdr8p, which regulates expression of the ABC efflux pumps (79). Therefore, *PDR8* directly regulates the same network as *PDR1* and thus would not represent an attractive therapeutic target for adjunctive therapy, as it would likely exert a similar selective pressure for resistance as a single therapy.

*HEK2* has a role in telomere maintenance alongside mRNA binding, localization, and stabilization (72, 73). Deletion of *CgHEK2* has previously been shown to increase susceptibility of *C. glabrata* to Caspofungin, implicating *CgHEK2* in response to multiple drug stresses (99). *CgHEK2* also contributes to the survival of *C. glabrata* within macrophages *in vitro,* with roles in oxidative stress resistance and modulation of ROS production within macrophages (100). This suggests that *CgHEK2* influences the ability of *C. glabrata* to respond to numerous environmental stresses and that inhibition of *CgHEK2* would alter the virulence of *C. glabrata,* alongside the slowing emergence of resistance.

*STE2* and *STE12* possess divergent roles in cellular processes. In *S. cerevisiae, STE2* encodes Ste2, a mating pheromone receptor in a-cells, which plays a role in the initiation of signaling cascades, resulting in mating (88, 89, 101, 102). Little is known about the contribution of *CgSTE2* to stress responses in *C. glabrata*; therefore, further elucidation of the role of *CgSTE2* in azole resistance is required. However, *CgSTE12* has characterized roles in both adaptation to stress and virulence in *C. glabrata* (103–105). *STE12* encodes a transcription factor, which regulates the expression of genes involved in mating, pseudohyphal, and invasive growth (92). Its ortholog*, CgSTE12,* regulates nitrogen starvation-induced pseudohyphae formation in *C. glabrata* (103). It was shown that *Cgste12Δ C. glabrata* mutants displayed attenuated virulence in murine models of candidiasis compared with WT and *CgSTE12* reconstituted strains (103). Additionally, *CgSTE12* possesses roles in biofilm formation and adaptation to environmental stresses, including osmotic, cell wall, oxidative, and genotoxic stresses (104, 105). Similar to *CgHEK2,* targeting *CgSTE12* should theoretically influence virulence, stress responses, and azole susceptibility within *C. glabrata*.

In *S. cerevisiae*, GCN5 is a component of the ADA and SAGA chromatin remodeling complexes (106). Similarly, in *C. glabrata*, CgGCN5 is part of the SAGA chromatin remodeling complex (94, 107). Specifically, *CgGCN5* encodes the lysine acetyltransferase CgGcn5p, which, alongside CgAda2p and CgAda3p, comprises the histone acetyltransferase (HAT) module within the SAGA complex (94, 107, 108).

*CgGCN5* plays a crucial role in drug resistance and virulence in *C. glabrata* (3, 94, 107–109). Its deletion increases sensitivity to various environmental stresses, including oxidative, cell wall, and antifungal stress (94, 108). Cggcn5Δ strains exhibit heightened susceptibility to azoles and echinocandins, reduced antifungal tolerance, and a diminished ability to acquire resistance (94, 108). Transcriptomic analysis has shown that GCN5 deletion alters the cell wall and reduces adhesion (108). Additionally, loss of CgGCN5 impairs biofilm formation, macrophage proliferation, and virulence in *G. mellonella* models (94, 108, 109). Orthologs of *CgGCN5* have also been linked to drug resistance in multiple fungal species, including *Candida* spp. (108, 110, 111), *Cryptococcus neoformans* (112, 113), and *Aspergillus fumigatus* (114). Given that disrupting *CgGCN5* slows the emergence of multidrug resistance, it represents an attractive target for adjunctive antifungal therapy.

A proof-of-concept study by Usher & Haynes (2019) demonstrated that deleting or inhibiting *CgGCN5* was lethal to *C. glabrata* cells carrying *CgPDR1^+^* alleles (3). Furthermore, it attenuated the emergence of Fluconazole resistance, validating the use of SGA screens to identify genetic interactions that influence drug resistance. However, this study used γ-butyrolactone, which is toxic to mammalian cells and unsuitable as an antifungal adjuvant (3, 60, 115). A separate study identified CPHT2 as a GCN5 inhibitor with fungicidal activity against multiple *Candida* species (111). However, CPHT2 does not affect *C. glabrata*, likely due to the absence of a specific histone H3 variant found in the CTG clade. Therefore, identifying clinically safe and effective *CgGCN5* inhibitors remains a critical need.

### *In silico* inhibitor identification

Using the HIP HOP database, potential inhibitors for the identified target gene were screened *in silico*. This approach facilitated the prioritization of compounds based on their Z-scores, indicating their theoretical efficacy in inhibiting the target gene (65). The identification of MTX as a potential inhibitor was particularly notable, given its previous use in various therapeutic contexts (95). The selection of MTX for further *in vitro* validation was informed by its promising *in silico* performance and identification of a study that described synergy between MTX and azoles against *C. albicans in vitro* (66).

### Toxicity screening

Use of *G. mellonella* as a model organism to screen toxicity of antifungals and other novel compounds is well-established (116–118). Toxicity screening within *G. mellonella* provided essential data on the cytotoxic effects of MTX. The survival curves indicated that MTX concentrations up to 0.32 mg/mL were well tolerated by the larvae (Fig. 3b), with higher concentrations showing increased toxicity. The Kaplan-Meier survival analysis reinforced the importance of careful dose management to balance antifungal efficacy and host safety.

### *In vitro* inhibitor validation

The spot assays and checkerboard screening provided robust data on the efficacy of MTX in combinations with FLZ. The results indicated a synergy between MTX and FLZ, enhancing the antifungal activity against *C. glabrata* strains, particularly those harboring various *CgPDR1*^+^ mutations. Although comparison across strain backgrounds introduces some inherent variability, our goal was to examine allele-specific effects in clinically relevant contexts. Thus, although the contribution of CgPDR1^R592S^ to azole resistance is supported, it cannot be assumed to be the sole determinant.

### Gene expression is affected by both MTX and FLZ exposure

We examined the differential gene expression in wild-type and DSY227 (CgPDR1^R592S^) *C. glabrata* cells under varying drug exposure conditions. Using Q-PCR, we assessed the expression of key regulatory genes, including *GCN5, STE12, STE2, HEK2*, and *PDR1*, using *PDA1* as a reference gene.

In wild-type cells, treatment with MTX resulted in an upregulation of *GCN5*, suggesting that MTX may modulate histone acetylation pathways through Gcn5p, a known histone acetyltransferase implicated in transcriptional regulation and stress responses in fungal pathogens (119). Conversely, exposure to fluconazole (FLZ) led to a significant upregulation of *PDR1*, consistent with the role of *PDR1* in mediating azole resistance by enhancing efflux pump expression (120). Notably, the combinatorial treatment of MTX and FLZ did not significantly alter the expression of any analyzed genes, indicating a possible counterbalancing effect that prevents major transcriptional shifts.

In DSY227 (*CgPDR1^R592S^*) mutant cells, basal (untreated) conditions showed an elevated expression of *PDR1*, aligning with previous findings that mutations in *PDR1* lead to constitutive activation and increased azole resistance (31). However, in MTX-treated DSY227 cells, we observed an increased expression of *GCN5* and *STE2*, with a concurrent decrease in *PDR1* expression. The increase in *GCN5* suggests an enhanced role of chromatin remodeling in response to MTX, while *STE2* induction may indicate altered MAPK signaling in response to stress (121). The downregulation of *PDR1* could suggest a potential negative regulatory mechanism induced by MTX in the resistant strain.

FLZ exposure in DSY227 cells resulted in the upregulation of *GCN5, STE2*, and *STE12*, with a decreased expression of *PDR1*. The increased expression of *GCN5* supports its involvement in stress adaptation and antifungal response (110). The upregulation of *STE2* and *STE12*, key genes in stress response pathways, could indicate a compensatory mechanism in response to drug-induced stress (19). The decrease in *PDR1* expression in the presence of FLZ is unexpected, as *PDR1* activation typically enhances azole resistance. This suggests that in the presence of the *CgPDR1^R592S^* mutation, alternative regulatory mechanisms might be at play, possibly due to feedback inhibition or compensatory shifts in stress response pathways (122).

Under combinatorial treatment (MTX + FLZ), DSY227 cells exhibited increased *GCN5* and *STE2* expression, whereas *PDR1* was downregulated. This pattern suggests that MTX and FLZ together may act synergistically to modulate chromatin remodeling gene expression, potentially altering cell stress responses and survival pathways (123). The decrease in *PDR1* suggests a unique regulatory interplay that diminishes its role in multidrug resistance under dual-drug conditions.

This demonstrates that gene expression responses in *C. glabrata* vary, depending on the genetic background and drug treatment conditions. The upregulation of *GCN5* across multiple conditions highlights the potential role of histone acetylation in drug response mechanisms. The unexpected decrease in *PDR1* expression in DSY227 under antifungal treatment suggests a complex regulatory network that requires further investigation.

### Pan-fungal screen with MTX and FLZ

The screening of multiple *Candida* species against FLZ and MTX, both individually and in combination, has provided a valuable insight into the potential antifungal strategy that could be employed by repurposing drugs. Our results show a differential susceptibility of *Candida* species to FLZ and MTX, also underscoring the complexity of antifungal resistance mechanisms in fungal pathogens. Consistent with the MIC data, FLZ at lower concentrations failed to inhibit the growth of the *Candida* species, aligning with known resistance profiles, especially in non-*albicans Candida* species, which often exhibit higher intrinsic resistance to FLZ (17, 61, 67, 96). At 32 µg/mL, a concentration above the clinical breakpoint, inhibition was observed in *C. glabrata, C. albicans,* and *C. dubliniensis*, indicating a partial susceptibility at this concentration (17, 61, 67, 96). However, full inhibition across all strains was only achieved at 128 µg/mL, considerably higher than typical therapeutic concentrations. This also highlights the limited efficacy of FLZ as a monotherapy against certain *Candida* species, such as *C. auris* and *C. parapsilosis*.

Our results indicate that MTX exhibits antifungal activity against all tested *Candida* species, complementing previous work on *C. albicans* (66). This suggests that MTX has a broad-spectrum antifungal effect, albeit with variable potency across the different species. The combination of FLZ and MTX showed synergy, as evidenced by the inhibition of growth of all tested *Candida* strains at relatively lower concentrations (8 µg/mL FLZ and 0.16 mg/mL MTX) at both 30°C and 37°C. The enhanced efficacy of the combination therapy compared with either compound alone suggests a synergistic interaction. This could be due to the combined effects of disrupting the fungal cell wall synthesis (by FLZ) and interfering with folate metabolism (by MTX), thereby attacking multiple pathways critical for fungal cell survival (95). The temperature-dependent variation in efficacy highlights the importance of considering physiological conditions when assessing antifungal therapies. The increased requirement of MTX at 37°C might be attributed to the higher metabolic rate or stress response mechanisms of the *Candida* species, reflecting the human host environment.

This study successfully demonstrated the utility of SGA screening combined with *in silico* and *in vitro* analyses to identify potential therapeutic targets and inhibitors for combating azole-resistant *C. glabrata* infections. The presence of the CgPDR1^R592S^ mutation aligns with high fluconazole resistance in the corresponding clinical isolate; additional genomic factors may contribute to this phenotype and cannot be excluded based on current data. Our findings are consistent with a model in which MTX modulates fluconazole susceptibility through interference with Gcn5-dependent transcriptional regulation; we acknowledge that MTX is a pleiotropic agent, and further work is necessary to define its precise mechanism of action in this context.

## SUPPLEMENTAL MATERIAL

Supplemental figures spectrum.02522-24-s0001.pdf Fig. S1 to S3.

Table S1 spectrum.02522-24-s0002.docx SGA Media and Media Prep.

Table S2 spectrum.02522-24-s0003.xlsx Genetic interaction hits from SGA screen.

ASM does not own the copyrights to Supplemental Material that may be linked to, or accessed through, an article. The authors have granted ASM a non-exclusive, world-wide license to publish the Supplemental Material files. Please contact the corresponding author directly for reuse.
