# Supplementary material for: Leveraging synthetic genetic array screening to identify therapeutic targets and inhibitors for combatting azole resistance in Candida glabrata
Source: Microbiol Spectr. 2025 Aug 11;13(9):e02522-24. doi: 10.1128/spectrum.02522-24 (PMC12403772; doi:10.1128/spectrum.02522-24)
Supplement: Supplemental figures — Fig. S1 to S3. [file spectrum.02522-24-s0001.pdf]

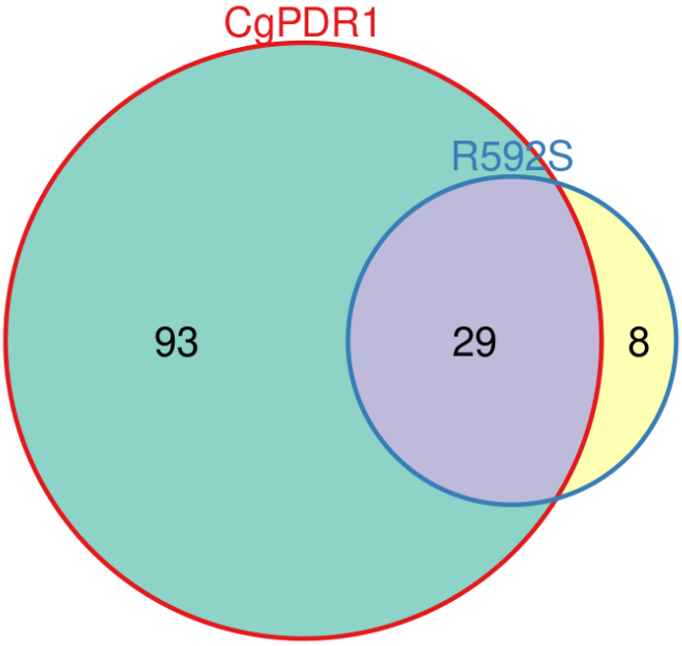

Supplemental Figure 1: A proportional Venn diagram of the number of SL and SS interactions shared by the WT *CgPDR1* and *CgPDR1*^{R592S} alleles. There were 93 interactions unique to the WT *CgPDR1* allele, 29 interactions were common to both alleles and 8 interactions were unique to the *CgPDR1*^{R592S} allele.

		Methotrexate (mg/mL)										
	Growth Con	MTX 0.002	MTX 0.005	MTX 0.01	MTX 0.02	MTX 0.04	MTX 0.08	MTX 0.16	MTX 0.32	MTX 0.64	MTX 1.28	Sterility Con
Fluconazole (ug/mL)	FLZ 4	Combi	Combi	Combi	Combi	Combi	Combi	Combi	Combi	Combi	Combi	Sterility Con
	FLZ 8	Combi	Combi	Combi	Combi	Combi	Combi	Combi	Combi	Combi	Combi	Sterility Con
	FLZ 16	Combi	Combi	Combi	Combi	Combi	Combi	Combi	Combi	Combi	Combi	Sterility Con
	FLZ 32	Combi	Combi	Combi	Combi	Combi	Combi	Combi	Combi	Combi	Combi	Growth Con
	FLZ 64	Combi	Combi	Combi	Combi	Combi	Combi	Combi	Combi	Combi	Combi	Growth Con
	FLZ 128	Combi	Combi	Combi	Combi	Combi	Combi	Combi	Combi	Combi	Combi	Growth Con
	FLZ 256	Combi	Combi	Combi	Combi	Combi	Combi	Combi	Combi	Combi	Combi	Growth Con

		Methotrexate (mg/mL)										
	Growth Con	MTX 0.04	MTX 0.08	MTX 0.16	MTX 0.32	MTX 0.64	MTX 1.28	MTX 2.56	MTX 5.12	MTX 7.68	MTX 10.24	Sterility Con
Fluconazole (ug/mL)	FLZ 4	Combi	Combi	Combi	Combi	Combi	Combi	Combi	Combi	Combi	Combi	Sterility Con
	FLZ 8	Combi	Combi	Combi	Combi	Combi	Combi	Combi	Combi	Combi	Combi	Sterility Con
	FLZ 16	Combi	Combi	Combi	Combi	Combi	Combi	Combi	Combi	Combi	Combi	Sterility Con
	FLZ 32	Combi	Combi	Combi	Combi	Combi	Combi	Combi	Combi	Combi	Combi	Growth Con
	FLZ 64	Combi	Combi	Combi	Combi	Combi	Combi	Combi	Combi	Combi	Combi	Growth Con
	FLZ 128	Combi	Combi	Combi	Combi	Combi	Combi	Combi	Combi	Combi	Combi	Growth Con
	FLZ 256	Combi	Combi	Combi	Combi	Combi	Combi	Combi	Combi	Combi	Combi	Growth Con

Supplemental Figure 2 Plate layout for checkerboard analysis

Plate layout for FLZ and MTX checkerboard at low and high concentrations

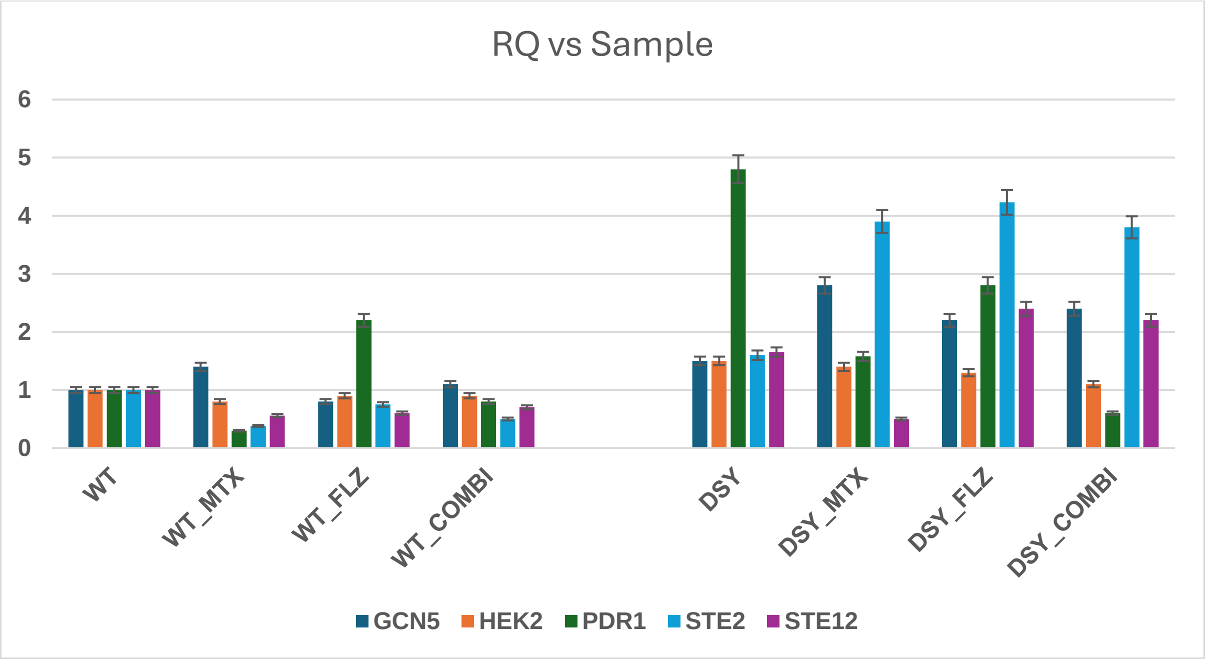

Supplemental Figure 3 Expression of genes that are in common for all gain-of-function PDR1 alleles.
