# Supplementary material for: Leveraging synthetic genetic array screening to identify therapeutic targets and inhibitors for combatting azole resistance in Candida glabrata
Source: Microbiol Spectr. 2025 Aug 11;13(9):e02522-24. doi: 10.1128/spectrum.02522-24 (PMC12403772; doi:10.1128/spectrum.02522-24)
Supplement: Table S1 — SGA Media and Media Prep. [file spectrum.02522-24-s0002.docx]

**Supplemental Table 1:**

**SGA Media and Media Prep**

SGA screening comprises of multiple steps to ensure that the final strains both express a *CgPDR1* allele (WT or *CgPDR1^+^*) and contain a single gene-deletion, to assay for SS or SL interactions. This is done by following a specific workflow, detailed in Usher, 2022, which utilises selective agar.

| **Step** | **Media** | **Recipe**  **(500mL)** | **Incubation Time (Days)** | **Incubation Temperature (°C)** |
| --- | --- | --- | --- | --- |
| Mating | YEPD + ADE | 0.06g of adenine, 5g of yeast extract (Y.E.), 10g of peptone, 10g of agar was mixed, and dissolved in 475mL of ddH_2_0. Following autoclaving of ingredients, add 25mL of 40% glucose. | 1 day | 22°C  (room temperature) |
| Diploid Selection | SD-URA LYS | 3.35g of yeast nitrogen base (Y.N.B.), 1g of drop-out mix (D.O. -URA LYS) is mixed and dissolved in 50mL of ddH_2_0. Separately, 10g of agar was mixed with 425mL of ddH_2_0. The drop-out and agar was mixed after autoclaving, and 25mL of 40% glucose was added. | 2 days | 30°C |
| Sporulation (SPO) | SPO + Y.E. + Glucose + AA | 5g of potassium acetate, 0.5g of yeast extract, 0.25g glucose, 0.05g amino acid add-back (which is a mix of 2g Ura, 2g His and 10g Leu) and 10g of agar was mixed, dissolved in 500mL of ddH_2_0 and autoclaved all together. | 14 days | 22°C (RT) |
| MATa Haploid (1^st^ round) | SD-HIS ARG + CAN | 3.35g of Y.N.B. without amino acids and 1g of drop-out mix (D.O. -HIS ARG) was mixed and dissolved in 50ml of ddH_2_0. Separately, 10g of agar was mixed with 425mL of ddH_2_0. After autoclaving, the drop out was mixed with agar, 25mL of 40% glucose and initially 0.25mL of 100mg/mL canavanine. However, this was later dropped to 0.25mL of 20mg/mL of canavanine. | 2 days | 30°C |
| MATa Haploid (2^nd^ round) | SD-HIS ARG + CAN | “ ” | 1 days | 30°C |
| kanR Selection | SD(msg)-HIS ARG + CAN, G418 | 0.85g of Y.N.B. without amino acids and ammonium sulfate, 0.5g of monosodium glutamic acid, 1g of drop-out mix (D.O. -HIS ARG) was mixed and dissolved in 50mL of ddH_2_0. Separately, 10g of agar was dissolved in 425mL of ddH_2_0. After autoclaving, the drop-out was mixed with agar, 50mL of 40% glucose was added, 0.5mL of 20mg/mL canavanine and 0.5mL of 200mg/mL of G418. | 2 days | 30°C |
| kanR URA Selection | SD(msg)-HIS ARG URA + CAN, G418 | 0.85g of Y.N.B. without amino acids and ammonium sulfate, 0.5g of monosodium glutamic acid, 1g of drop-out mix (D.O. -HIS ARG URA) was mixed and dissolved in 50mL of ddH_2_0. Separately, 10g of agar was dissolved in 425mL of ddH_2_0. After autoclaving, the drop-out was mixed with agar, 50mL of 40% glucose was added, 0.5mL of 20mg/mL canavanine and 0.5mL of 200mg/mL of G418. | 1-2 days | 30°C |

**Supplemental Methods 1:**

#install and call packages

install.packages('gplots', repos='https://www.stats.bris.ac.uk/R/')

library('gplots')

#Create data frame

flz <- c('FLZ 0', 'FLZ 4', 'FLZ 8', 'FLZ 16', 'FLZ 32', 'FLZ 64', 'FLZ 128', 'FLZ 258')

mtx <- c('MTX 0', 'MTX 0.04', 'MTX 0.08', 'MTX 0.16', 'MTX 0.32', 'MTX 0.64', 'MTX 1.28', 'MTX 2.56', 'MTX 5.12', 'MTX 7.68', 'MTX 10.24')

df.cw <- read.csv(file.choose(), header = TRUE, row.names = flz, col.names = mtx)

df.cw

# Mutate any growth value <0 to be 0

df.cw[df.cw < 0] <- 0

df.cw.m <- as.matrix(df.cw)

df.cw.m

#visualise dataset

heatmap.2(df.cw.m, Colv = FALSE, Rowv = FALSE,

scale = 'none', margins = c(8,8),

trace = 'none',

density = 'none',

xlab = 'MTX concentration (mg/mL)', ylab = 'Fluconazole concentration (μg/ml)',

symm=F, symkey = FALSE, symbreaks = TRUE,

key.title = 'Growth',

key.xtickfun = function() {

breaks = pretty(parent.frame()$breaks)

breaks = breaks[c(1,length(breaks))]

list(at = parent.frame()$scale01(breaks),

labels = breaks)},

keysize = 1.1,

breaks = seq(0,1,0.01), # seq( lower limit, upper limit, interval)

col = colorpanel(100, 'black', 'green') # colours as the scale -> colorpanel( number of colours, lower value colour, upper value colour)

)
